# Targeted photodynamic therapy of cancer using a novel gallium (III) tris (ethoxycarbonyl) corrole conjugated‐mAb directed against cancer/testis antigens 83

**DOI:** 10.1002/cam4.1601

**Published:** 2018-06-01

**Authors:** Ziyu Ye, Yanfang Liang, Yan Ma, Bihua Lin, Longbin Cao, Bin Wang, Zhao Zhang, Haibo Yu, Jixia Li, Mingyuan Huang, Keyuan Zhou, Qunzhou Zhang, Xinguang Liu, Jincheng Zeng

**Affiliations:** ^1^ Dongguan Key Laboratory of Medical Bioactive Molecular Developmental and Translational Research Guangdong Provincial Key Laboratory of Medical Molecular Diagnostics Guangdong Medical University Dongguan China; ^2^ Department of Pathology The Fifth People's Hospital of Dongguan Dongguan Hospital Affiliated to Medical College of Jinan University Dongguan China; ^3^ Collaborative Innovation Center for Antitumor Active Substance Research and Development Guangdong Medical University Zhanjiang China; ^4^ Department of Chemistry South China University of Technology Guangzhou China; ^5^ Department of Oral and Maxillofacial Surgery and Pharmacology University of Pennsylvania School of Dental Medicine Philadelphia PA USA; ^6^ Institute of Aging Research Guangdong Medical University Dongguan China

**Keywords:** cancer/testis antigens 83, Gallium (III) 5,10,15‐tris (ethoxycarbonyl) corrole, hybridoma, monoclonal antibodies, photodynamic therapy

## Abstract

Photodynamic therapy (PDT) is a noninvasive, highly selective approach to the treatment of tumors. However, its therapeutic effect is limited by long‐lasting skin phototoxicity. Therefore, to compromise this shortcoming, it is preferable to deliver photosensitizers selectively to tumor cells with the aid of antibodies specific against tumor‐associated antigens. Cancer/testis antigens 83 (CT83), also called KK‐LC‐1 or CXorf61, recognized by cytotoxic T lymphocytes (CTL), has become a promising target for immunotherapy. Herein, we developed and characterized a novel mouse CT83 mAb 7G4 with a high affinity with Gallium (III) 5, 10, 15‐tris (ethoxycarbonyl) corrole (1‐Ga), a new and promising photosensitizer in PDT. The enzyme‐linked immunosorbent assay (ELISA), flow cytometry and cytotoxicity activity assays revealed that 7G4‐1‐Ga was able to recognize human CT83 with high specificity. Furthermore, 7G4‐1‐Ga showed greater cytotoxicity to CT83‐expressing human cancer cells in vitro than 1‐Ga. These results suggest that the antibody‐conjugated photosensitizer between anti‐CT83 mAb and 1‐Ga may have a good application in PDT, where the destruction of CT83‐expressing tumor is required.

## 1 INTRODUCTION

Photodynamic therapy (PDT) is a minimally invasive, highly selective approach to the treatment of various malignant tumors and non‐malignant diseases.^1^ However, its therapeutic efficacy is limited by long‐lasting skin phototoxicity. The trigger of PDT requires the simultaneous presence of three components, photosensitizer, molecular oxygen, and visible light. One of the intrinsic drawbacks of the “first generation” photosensitizers was its long half‐life and prolonged retention in normal cells.^2^ In recent years, much effort has been devoted to develop new generations of photosensitizers. A corrole as a ring‐contracted analogue of porphyrin has been intensively investigated as photosensitizers in PDT. Our previous study showed that Gallium (III) 5, 10, 15‐tris (ethoxycarbonyl) corrole (1‐Ga) could rapidly penetrate the cell membrane and exhibit remarkable phototoxicity, suggesting its potential as an ideal photosensitizer.^3^ However, similar to other photosensitizers, its therapeutic efficacy is limited by long‐lasting skin phototoxicity. To overcome such shortcomings, it is preferable to deliver photosensitizers selectively to tumor cells.

Cancer/testis (CT) antigens, a group of tumor‐associated antigens, are specifically expressed in testis and placenta but not in other normal tissues, whereas they are found to be aberrantly activated and expressed in various types of human cancers.^4^, ^5^ Recently, several CT antigens such as members of melanoma‐associated antigen (MAGE) family and New York Esophageal Squamous Cell Carcinoma‐1 (NY‐ESO‐1) have been found to induce spontaneous humoral and cell‐mediated immune responses in cancer patients.^6^, ^7^, ^8^, ^9^, ^10^ CT antigens 83 (CT83), also called Kita‐Kyushu lung cancer antigen‐1 (KK‐LC‐1) or CXorf61, recognized by cytotoxic T lymphocytes (CTL), has become a promising immunotherapy target.^11^ Several studies have reported that CT83 is highly expressed in non‐small cell lung cancer (32.6%),^12^ triple negative breast cancer (75%),^13^ gastric cancer (81.6%),^14^ colorectal cancer (62.5%), and nasopharyngeal cancer (90.2%) patients (our unpublished data). The Human Protein Atlas (http://www.proteinatlas.org) data‐sets also revealed that *CT83* transcripts were expressed in lung cancer, stomach cancer, colorectal cancer, urothelial cancer, cervix cancer, breast cancer, and various tumor cell lines (U‐266/70, HeLa, U‐266/84, K‐562, U‐2OS). Hence, CT83 may be a potential target for antibody‐photosensitizer conjugate‐based PDT to a variety of malignancies.

Herein, we developed and characterized a novel mouse monoclonal antibody specific against human CT83 (CT83 mAb 7G4), which can effectively bind to 1‐Ga to form antibody‐photosensitizer complex 7G4‐1‐Ga. The enzyme‐linked immunosorbent assay (ELISA), flow cytometry, and cytotoxicity activity assays revealed that 7G4‐1‐Ga was able to recognize human CT83 with high specificity. Furthermore, 7G4‐1‐Ga showed greater cytotoxicity to CT83‐expressing human cancer cells in vitro than that of 1‐Ga. These results suggest that the antibody‐conjugated photosensitizer, anti‐CT83 mAb and 1‐Ga, might be a promising targeted PDT for effective treatment of CT83‐expressing tumors.

## 2 MATERIALS AND METHODS

### 2.1 Materials

Freund's complete and incomplete adjuvants, HAT (hypoxanthine, aminopterin, thymidine) medium, and polyethylene glycol 1500 (PEG1500), were purchased from Sigma‐Aldrich (St. Louis, MO, USA). Soluble human CT83 protein prokaryotic expression system (pET28a‐CT83 in *E. coli* BL21 (DE3)) was constructed in our laboratory. The SBA Clonotyping (IgA, IgG1, IgG2a, IgG2b, IgG3, IgM, λ, κ) System‐HRP kit was purchased from Southern Biotech (USA). Gallium(III) 5,10,15‐tris (ethoxycarbonyl) corrole (1‐Ga) was obtained from Key Laboratory of Functional Molecular Engineering of Guangdong Province, South China University of Technology, and prepared according to the literature.^3^ 6 weeks old, female BALB/c mice were purchased from the Guangdong Animal Research Institute (Guangzhou, China). All animals were strictly handled according to the Good Animal Practice Requirements of the Animal Ethics Procedures and Guidelines of the People's Republic of China. The present study was approved by the Animal Ethics Committee of the Dongguan Key Laboratory of Medical Bioactive Molecular Developmental and Translational Research, Guangdong Medical University (Approval no. 20150007).

Dulbecco's Modified Eagle Medium (DMEM) and fetal bovine serum (FBS) were purchased from Gibco (Grand Island, NY, USA). Human colorectal cancer cell lines (RKO, SW1116), lung cancer cell line (NCI‐H1299), human hepatoma cell line (HuH‐7), cervix cancer cell line (HeLa), breast cancer cell lines (MCF‐7, MDA‐MB‐231), nasopharyngeal cancer cell lines (HNE‐1, CNE‐2), embryonic kidney cell line (293T) and Sp2/0‐Ag14 cell line were obtained from the Cell bank of Chinese Academy of Sciences (Shanghai, China). Human lung cancer, nasopharyngeal cancer, cervix cancer, colorectal cancer, breast cancer, gastric cancer, melanoma, urothelial carcinoma, and endometrioid adenocarcinoma tissue sections were acquired from the Fifth People's Hospital of Dongguan under an approved IRB and informed consent was obtained from all human subjects before the study.

### 2.2 CT83 epitope prediction

According to CT83 primary structure reported from the NCBI Guide at http://www.ncbi.nlm.nih.gov, the secondary structure, surface probability, flexibility, and antigenicity of the CT83 were analyzed by DNAStar software. Amino acid sequences with β‐turn or random‐coil, and few α‐helix and β‐sheet, good hydrophilicity, high accessibility, high flexibility, and strong antigenicity were selected as protein antigen epitope sequences.

### 2.3 Synthesis of genes encoding CT83 antigen fragments

According to the CT83 epitope prediction results, the best epitope sequence was selected and synthesized by gene synthesis technology. The synthesized gene sequence also was amplified by PCR. The primers for PCR were as follows: 5′‐CTGGATCCTATCGTCGTTTTCAGCGAAAC‐3′ (forward); 5′‐GCCTCGAGTTAGGTACTTTTACGATGCG‐3′ (reverse). Conditions for PCR amplification were 5 minutes predenaturation at 94°C and followed by 30 cycles of 30 seconds denaturation at 94°C, 30 seconds annealing at 54°C and 30 seconds extensions at 72°C, and for 5 minutes at 72°C for final extension. The amplified gene sequence was inserted into the pET28a vector at restriction enzyme sites B*am*H I and X*ho* I.

### 2.4 Preparation of recombinant human CT83 proteins

The plasmid pET28a vector and the target gene were digested by corresponding endonucleases B*am*H I and X*ho* I, respectively. The fragments were ligated by T(4) DNA ligase to gain recombinant expression vector. Then, the recombinant plasmid pET28a‐CT83 was electro‐transformed into BL21 (DE3) strain, and expressed in the bacteria under induction of 0.5 mmol/L isopropyl β‐D‐thiogalactoside (IPTG), at 20°C for 12 hours. After this, the bacteria were centrifuged at 5000 *g*, at 4°C for 15 minutes. The harvested bacteria were resuspended in phosphate buffer saline (PBS) and lysed by ultrasonication, and the lysate was centrifuged at 10621 g at 4°C for 10 minutes. The supernatant and pellets of the lysate were analyzed using 12% SDS‐PAGE.

### 2.5 Production of CT83 mAb and validation of antigen specificity

Recombinant human CT83 proteins were purified with a Nickel ion nitrilotriacetic acid column (GE Healthcare) and eluted with different concentrations of imidazole (10, 20, 50, 200, 500 mmol/L), then confirmed through a 12% sodium dodecyl sulfate polyacrylamide gel electrophoresis (SDS‐PAGE) gel stained with coomassie brilliant blue. The total protein concentrations in the pellets were determined by 12% SDS‐PAGE and BCA protein assay kit (Beyotime, China). BALB/c mice were immunized biweekly by intraperitoneal and subcutaneous injections with 50 μg purified CT83 protein in freund's adjuvant. Booster injections of 25 μg CT83 protein without adjuvant were administered intravenously 3 days prior to the cell fusion. Splenic lymphocytes were fused with mouse SP2/0 cells and hybridomas selected in DMEM medium supplemented with hypoxanthine, aminopterin, and thymidine. Hybridomas secreting CT83 mAbs were selected by indirect ELISA. Hybridoma supernatant containing mAbs that reacted with *E. coli* CT83 only were selected for limiting dilution and further characterization. The monoclonal antibodies were purified from hybridoma supernatant by Protein A agarose chromatography then the purified antibodies were conjugated with HRP by using peroxidase labeling kit (Roche, Germany) according to the manufacturer's instructions. Immunoglobulin isotypes were determined using a mouse SBA Clonotyping (IgA, IgG1, IgG2a, IgG2b, IgG3, IgM, λ, κ) System‐HRP kit (SouthernBiotech, Birmingham, USA), according to the manufacturer's instructions.

### 2.6 ELISA analysis

Briefly, 96‐well microtiter plates were coated overnight at 4°C with 100 μL/well purified *E. coli* CT83 protein (2 μg/mL). After washed three times with PBST (0.05% Tween‐20 in PBS), plates were blocked with PBS containing 1.0% bovine serum albumin and washed with PBST. Undiluted hybridoma culture supernatant (100 μL/well) was added, incubated with agitation at room temperature for 1 hour, and washed with PBST. Bound mAbs were detected using HRP‐conjugated goat‐anti‐mouse IgG secondary antibodies (1:5000), 3,3′,5,5′‐tetramethylbenzidine (TMB) substrate, and H_2_SO_4_ (2 mmol/L). Optical density at 450 nm (OD 450) was determined by a microplate reader (Bio‐Rad, Richmond, CA, USA).

### 2.7 Western blot analysis

The prokaryotic expressed human CT83 protein as control and the endogenous CT83 protein of cancer cells was separated on a 12% SDS‐PAGE gel at 160 V for 60 minutes and transferred to 0.45 μm PVDF membrane at 100 V for 60 minutes. The membrane was blocked at room temperature for 60 minutes in TBST with containing 5% skim milk. Subsequent to being washed with TBST three times, the membrane was incubated with mAb against CT83 (1:1000) overnight for 4°C. The membrane was washed with TBST three times and incubated with goat anti‐mouse IgG HRP conjugated secondary antibody (1:5000) for 60 minutes at room temperature. The membranes were washed with TBST three times. The proteins were detected by a chemiluminescence detection system.

### 2.8 Immunohistochemistry analysis

Tissue sections (4 μm) were prepared from paraffin blocks. For immunostaining, the sections should be baked, deparaffinized, and rehydrated, antigenic epitopes retrieval was achieved by heating for 10 minutes in 10 mmol/L citrate buffer (pH 6.0) in a pressure cooker. Then the slides were incubated in 3% H_2_O_2_ solution for 10 minutes at room temperature to block the endogenous peroxidase activity. The slides were then incubated overnight with the mAb against CT83 for at 4°C, followed by HRP‐conjugated secondary detection antibody and diaminobenzidine (DAB). Immunoreactivities for the CT83 positive expression were defined by the cytoplasm appearing as brown granules.

### 2.9 Preparation of 7G4‐1‐Ga

Gallium (III) 5,10,15‐tris(ethoxycarbonyl)corrole (1‐Ga) was prepared according to the literature.^3^ A solution of 1‐Ga (10 mg, 0.017 mmol) and 5 mL antibody (1.5 mg/mL, PBS) in antibody diluent (DMSO: Tween: Diluent = 1:2:97, 15 mL, Beyotime) was stirred and re‐fluxed for 12 hours in ice. The resultant solution was evaporated to 5 mL through vacuum freeze drying. The mix complex 7G4‐1‐Ga was separated and purified by column chromatography on alumina. All procedures were carried out in the dark. The amount 1‐Ga bound to 7G4‐1 was determined by measuring the absorbance at 400 nm. And the protein concentration was determined by measuring the absorbance at 280 nm. Then, the 7G4‐1‐Ga was used in all bio‐related testing.

### 2.10 Cytotoxicity activity assay

The cytotoxicity activity of 7G4‐1‐Ga to cancer cells was measured by MTT assay.^3^ Briefly, 5 × 10^3^ of cells were seeded in 96‐well tissue culture plates for 24 hours. Cells incubated with 7G4‐1‐Ga without illumination (dark control) were kept in parallel for 1 hour. Cells were washed with PBS without illumination. After washing, 20 μL per well of MTT solution (5.0 mg/mL) was added and incubated for 4 hours. The medium was aspirated and replaced with 100 μL per well DMSO to dissolve the formazan salt. Absorbance was measured at 450 nm with 650 nm used as the reference wavelength.

### 2.11 Flow cytometric analysis

1 × 10^6^ of CT83‐expressing and CT83‐non‐expressing cells were collected, and incubated with 5ug/mL 7G4 or an equivalent dose of 7G4‐1‐Ga for 30 minutes. Then cells were stained with PE‐conjugated anti‐mouse IgG (MOPC‐21, BioLegend, USA) for 30 minutes after wash twice with PBS. Finally, CT83‐positive cells were analyzed by flow cytometry (BD FACS Calibur II, San Jose, CA, USA) as we previously described.^15^, ^16^ Flow cytometry data were analyzed using FlowJo 7.6 software (TreeStar Inc., USA).

### 2.12 Statistical analysis

Statistical analyses were performed as previously described using SPSS 20 statistical software.^17^, ^18^, ^19^ Each sample was analyzed in triplicate using an ELISA and a cell proliferation assay. The differences in mean values between control and samples treated with different concentrations of CT83 or CT83 mAbs were analyzed using the *t* test and variations considered significant at *P *<* *.05.

## 3 RESULTS

### 3.1 Preparation of recombinant human CT83 protein

**Figure 1:**
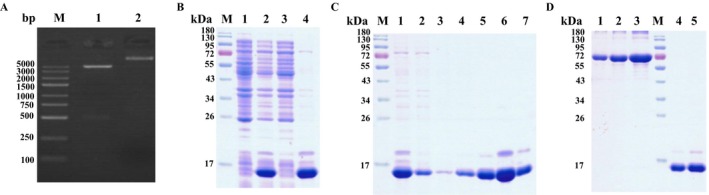
Recombinant human CT83 determination. A, Purified recombinant plasmid pET28a‐CT83 (lane 2), and digested by Bgl II and X*ho* I (lane 1). B, The recombinant pET28a‐CT83 plasmid was transformed into E.coli BL21 (DE3) and a 14 kDa recombinant protein was induced by 0.5 mmol/L IPTG (lane 2), compared with the uninduced group (lane 1). And most of recombinant protein was detected on pellet (lane 4) but not supernatant (lane 3) of ultrasonic crushing fluid. C, Recombinant protein was purified by affinity chromatography. Lane 1: ultrasonic precipitation solution dissolved with Urea. Lane 2: flow liquid. Lane 3: 10 mmol/L imidazole. Lane 4: 20 mmol/L imidazole. Lane 5: 50 mmol/L imidazole. Lane 6: 200 mmol/L imidazole. Lane 7: 500 mmol/L imidazole. D, Determination of recombinant protein concentration by SDS‐PAGE. Lane 1: 1 μg BSA standard. Lane 2: 2 μg BSA standard. Lane 3: 4 μg BSA standard. Lane 4: 1 μL CT83 recombinant protein. Lane 5: 2 μL CT83 recombinant protein. M: Molecular weight of DNA (bp) or protein (kDa) marker

The synthesized target gene sequence encoding ideal CT83 epitopes was subcloned into pET28a (+) plasmid digested by BamH I and Xho I enzymes before confirming with restriction enzyme analysis (Figure 1A) and DNA sequencing. Then the recombinant plasmid was transformed into E.coli BL21 (DE3) to induce protein expression with 0.5 mmol/L IPTG. SDS‐PAGE analysis revealed that the purified protein had a molecular weight of 14 kDa (Figure 1B), consistent with the predicted molecular weight of the recombinant protein. Immobilized metal ion affinity chromatography (IMAC) was conducted to purify the target protein. SDS‐PAGE analysis revealed that the target proteins were eluted easily in the range of 50‐500 mmol/L imidazole (Figure 1C) and the concentration of the purified protein was 1.0 mg/mL (Figure 1D).

### 3.2 Generation of antihuman CT83 mAbs

To create monoclonal antibodies against CT83, BALB/c mice were immunized with recombinant human CT83 protein, and hybridomas were generated by using single‐cell‐cloned method. The hybridomas were screened for their activity of binding to recombinant human CT83 protein by indirect ELISA, resulting in the establishment of three clones (7G4, 7A10, 7B4) expressing mice anti‐CT83 mAbs. The class and subclass of the selected clones and antibody titers in the cell culture supernatant of the clones were summarized in Table 1. From the results, the 7A10 cell line belonged to IgG2a subtype and the 7B4 and 7G4 cell lines belonged to IgG1 subtype, all of which have the kappa (κ) light chain.

**Table 1:** Characterization of selected clones in terms of titer, Ig class, and Ig subclass

Hybridoma	Class and subclass	Titer of supernatant of cell culture medium
7G4	IgG1, κ	51 200
7A10	IgG2a, κ	25 600
7B4	IgG1, κ	25 600

### 3.3 Purity and titer of purified antihuman CT83 mAb 7G4

**Figure 2:**
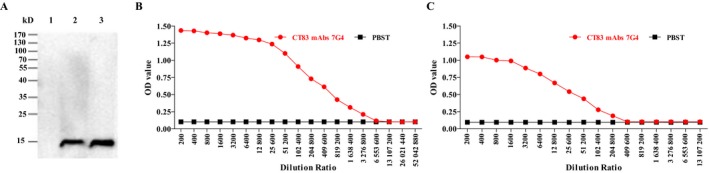
Purity and titer analysis of purified CT83 mAbs 7G4. To create monoclonal antibodies against CT83, BALB/c mice were immunized with recombinant human CT83 protein, and hybridoma cell 7G4 was generated by using the iliac lymph node method. The hybridomas were screened for their activity to bind recombinant human CT83 protein by indirect ELISA. A, a SDS‐PAGE determinant of 7G4 antibody. Lane 1: 0 μL purified CT83 mAb 7G4. Lane 2: 5 μL purified CT83 mAb 7G4. Lane 3: 10 μL purified CT83 mAb 7G4. B, Titer detection of before purification CT83 mAb 7G4 in ascites. C, Titer detection of purified CT83 mAb 7G4

As antihuman CT83 mAb 7G4 had the highest titer among all clones, it was selected to produce mAb. Protein A Sepharose column purification resulted in a concentration of 1.5 mg/mL for the mAb. SDS‐PAGE analysis showed one bands with molecular weight of 14 kDa (Figure 2A). Indirect ELISA analysis showed that the titer in ascites was 3.28 × 10^6^ (Figure 2B), and the titer for the purified antibody was 2.048 × 10^5^ (Figure 2C).

### 3.4 Characterization of the specificity of the purified antihuman CT83 mAb 7G4

**Figure 3:**
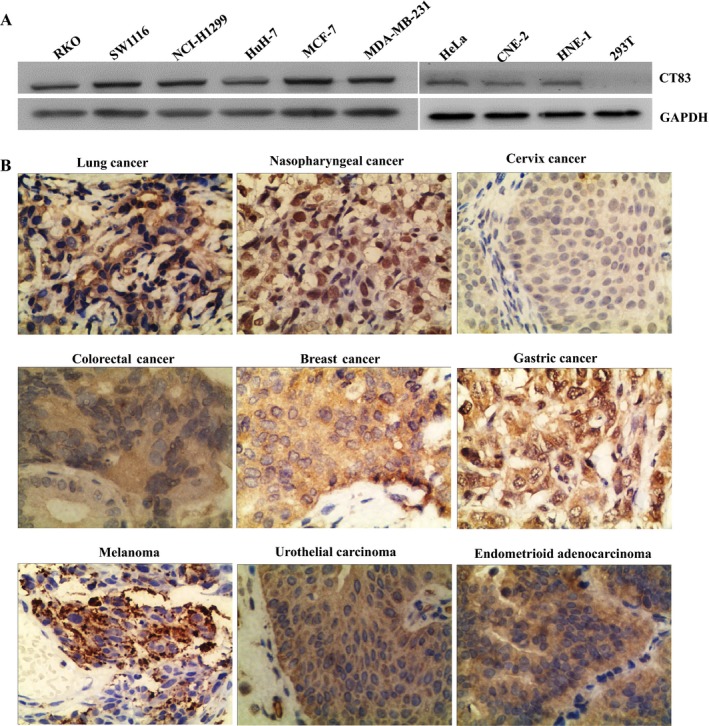
Identification of the specificity of the purified CT83 mAb 7G4 by Western blotting and immunohistochemistry. A, Western blotting was employed to validate the specificity of CT83 mAb 7G4 in detection of endogenous human CT83 in RKO, SW1116, NCI‐H1299, HuH‐7, HeLa, MCF‐7, MDA‐MB‐231, HNE‐1, CNE‐2 cancer cell lines, and 293T cell line. B, Immunohistochemistry was employed to validate the specificity of CT83 mAb 7G4 in detection of endogenous human CT83 in lung cancer, nasopharyngeal cancer, cervix cancer, colorectal cancer, breast cancer, gastric cancer, melanoma, urothelial carcinoma, and endometrioid adenocarcinoma tissue (400×)

Western blotting analysis showed that purified antihuman CT83 mAb 7G4 could specifically recognize the recombinant CT83 protein and the endogenous CT83 protein of the RKO, SW1116, NCI‐H1299, HuH‐7, HeLa, MCF‐7, MDA‐MB‐231, HNE‐1, CNE‐2 cancer cell lines with molecular weight of about 14 kDa, which was corresponding to the molecular weight of recombinant CT83 protein (Figure 3A). Meanwhile, immunohistochemical staining showed that purified antihuman CT83 mAb 7G4 could react with endogenous human CT83 expressed in lung cancer, nasopharyngeal cancer, cervix cancer, colorectal cancer, breast cancer, gastric cancer, melanoma, urothelial carcinoma, and endometrioid adenocarcinoma tissues, whereby the positive immune reactive signals were located on cell membranes and in cytoplasm (Figure 3B).

### 3.5 Binding activity of 7G4‐1‐Ga to purified CT83 and CT83‐expressing cells

**Figure 4:**
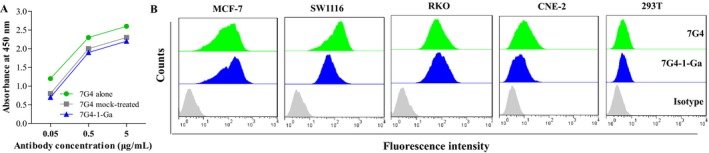
CT83 binding activity of 7G4‐1‐Ga was analyzed by ELISA and flow cytometry. A, The reactivity of mAb 7G4, 7G4‐1‐Ga complex with CT83 was estimated by ELISA using CT83 coated plates. B, The CT83‐expressing cancer (MCF‐7, SW1116, RKO, CEN‐2) cells and CT83‐non‐expressing 293T cell were incubated with 7G4, 7G4‐1‐Ga, then with PE‐conjugated anti‐mouse IgG before flow cytometry analysis

**Figure 5:**
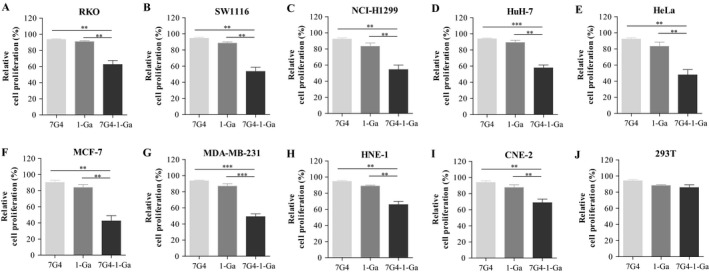
The cytotoxicity of CT83‐expressing cells following 7G4‐1‐Ga treatment. The cytotoxicity to CT83‐expressing cells in vitro were incubated for 1 hour with 1 μmol/L 1‐Ga or 5 μg/mL 7G4‐1‐Ga containing equivalent amounts of 1‐Ga, washed twice, then given PDT. As a result, in this condition, pretreatment with 1‐Ga slightly increased the killing activity. However, more than 20% ~ 60% of the cells were killed after incubation with 7G4‐1‐Ga. Values were represented as the mean ± standard error of the mean (SEM). **P <* *.01 and ***P <* *.001

The binding activity of 7G4 and 7G4‐1‐Ga to purified CT83 was determined by ELISA. Although the binding activity was reduced by conjugation with 1‐Ga, 7G4‐1‐Ga still exhibited a high level of binding activity over the concentration range 0.5‐5 μg/mL (Figure 4A). The reactivity with CT83‐expressing cells was examined by flow cytometry. 7G4‐1‐Ga also had a similar strong binding activity to the CT83‐expressing cells with 7G4 (Figure 4B). Our previous research found that 1‐Ga could induce cell apoptosis and inhibit cell proliferation upon photodynamic therapy (PDT) treatment in lung cancer^3^ and colorectal cancer cells (unpublished data). Therefore, the in vitro cytotoxicity of 7G4‐1‐Ga to CT83‐expressing cells was evaluated. To this purpose, cells were incubated for 1 hour with 1 μmol/L 1‐Ga or 7G4‐1‐Ga containing equivalent amounts of 1‐Ga, washed twice, and then given PDT. As a result, in this condition, pretreatment with 1‐Ga slightly increased the killing activity. However, more than 20% ~ 60% of the cells were killed after incubation with 7G4‐1‐Ga (Figure 5). These results suggested that 7G4‐1‐Ga can effectively and specificity binds to the surface of CT83‐expressing cancer cells via targeting CT83 on cancer cell surface mediated by CT83 mAb 7G4.

## 4 DISCUSSION

Due to the strong immunogenicity and specificity, CT antigens are considered as ideal targets for cancer immunotherapy, including therapeutic cancer vaccines and adoptive T‐cell transfer with chimeric T‐cell receptors.^20^, ^21^ Meanwhile, studies have also shown that CT antigens play important roles in oncogenesis, including immortality, invasiveness, hypo‐methylation, metastatic capacity, and immune evasion.^21^, ^22^, ^23^

CT83 with the MAGE family (MAGE‐A1, MAGE‐A3, MAGEH1, MAGE‐A9, MAGE‐C1/CT7, and MAGE‐C2/CT10) and NY‐ESO‐1, the most prevalently expressed antigens in spontaneous humoral to mediate T cell responses, has been described in tumor patients.^24^, ^25^, ^26^, ^27^, ^28^ Shida et al^14^ reported that CT83 expression was observed in gastric cancer, while the frequency of CT83 expression (81.6%) was higher than that of the MAGE‐A1 (34.7%), MAGE‐A3 (44.9%), and NY‐ESO‐1(16.3%). It was worth noting that even in patients with stage I gastric cancers, the CT83 expression rate (79.4%) was high, suggesting that CT83 was a potential biomarker for early diagnosis of gastric cancer.^29^ Additionally, Fukuyama et al^30^ reported that anti‐H. Pylori IgG titers from the CT83‐positive gastric cancer patients were significantly higher (67.5 ± 7.6) than those from CT83‐negative gastric cancer patients (15.8 ± 7.5). Immune therapy targeting CT antigens can improve the survival in several types of solid tumors. The expression of CT83 was related to poor survival in non‐small‐cell lung cancer (NSCLC).^12^, ^31^ In a study of triple‐negative breast cancer, the expression rate of CT83 was reported to be as high as 75%, which is related to lymph node metastasis, lymphatic invasion, and disease stage.^13^ In our study, we found that the expression rate of CT83 was as high as 62.5% and 90.2% in colorectal cancer and nasopharyngeal carcinoma (unpublished data), respectively.

CT83 had oncogenic functions, including support of growth, survival, and metastasis, and then it could be a useful target for cancer immunotherapy. However, there have been few commercially available mAbs against CT83 so far. In the current study, monoclonal antibodies against human CT83 were developed by fusing solenocytes from immunized mouse with SP2/0 myeloma cells and polyethylene glycol. Then the antibodies were screened with prokaryotic expressed human CT83 protein and the endogenous CT83 protein of the cancer cell lines subsequently. Western blotting, immunohistochemistry and cell proliferation assay mediated by 7G4‐1‐Ga revealed that selected CT83 mAb 7G4 were able to recognize human CT83 with high specificity. More importantly, the CT83 mAb 7G4 was a unique neutralizing mAb that suppressed CD8^+^ T cell proliferation with allogeneic mixed lymphocyte reaction (unpublished data). Previously, we found that 1‐Ga showed stronger PDT phototoxicity against cancer cells.^3^ In the present study, we developed and characterized a novel mouse CT83 mAb 7G4 to enhance 1‐Ga phototoxicity on CT83‐expressing cancer cells. Thus, CT83 mAb 7G4 might be a good candidate in the development of new targeted PDT for effective treatment of CT83‐expressing cancers.

## CONFLICT OF INTEREST

The authors have no conflicts of interest to declare.
